# Retraction Note: Psycho-linguistic and educational challenges in teaching Chinese (Mandarin) language: voices from none-Chinese teachers of Mandarin language

**DOI:** 10.1186/s40359-025-02818-6

**Published:** 2025-05-06

**Authors:** Lei Pan, Dan Sun, Yumei Zou, Yi Cao, Jingxian Zhang, Fangfang Li

**Affiliations:** 1 School of International Education, Hainan Medical University, Haikou City, 571199 Hainan Province China; 2 Hainan Normal University, No. 99, Longkun South Road, Qiongshan District, Haikou, Hainan China; 3 School of Foreign Languages, Jiangxi Agricultural University, Jiangxi, China; 4 Guangxi Normal University, Guangxi, China; 5 Hainan Medical University, Hainan, China; 6 School of Humanities, Shangqiu Normal University, Shangqiu, Henan, 476000 China

**BMC Psychology (2023) 11:390**

10.1186/s40359-023-01432-8

The editor and the publisher have retracted this article. The article was submitted to be part of a guest-edited issue. An investigation by the publisher found a number of articles, including this one, with a number of concerns, including but not limited to compromised editorial handling and peer review process, inappropriate or irrelevant references or not being in scope of the journal or guest-edited issue. Based on the investigation’s findings the editor therefore no longer has confidence in the results and conclusions of this article.

Pan Lei disagree has stated that the authors disagree with this retraction.

